# A Survey of Stimulation Methods Used in SSVEP-Based BCIs

**DOI:** 10.1155/2010/702357

**Published:** 2010-03-07

**Authors:** Danhua Zhu, Jordi Bieger, Gary Garcia Molina, Ronald M. Aarts

**Affiliations:** ^1^Department of Signal Processing Systems, Technical University Eindhoven, 5600 MB Eindhoven, The Netherlands; ^2^Department of Brain, Body & Behavior, Philips Research Eindhoven, 5656 AE Eindhoven, The Netherlands; ^3^College of Biomedical Engineering and Instrument Science, Zhejiang University, 310027, China; ^4^Department of Artificial Intelligence, Radboud University Nijmegen, 6500 HE Nijmegen, The Netherlands

## Abstract

Brain-computer interface (BCI) systems based on the steady-state visual evoked potential (SSVEP) provide higher information throughput and require shorter training than BCI systems using other brain signals. To elicit an SSVEP, a repetitive visual stimulus (RVS) has to be presented to the user. The RVS can be rendered on a computer screen by alternating graphical patterns, or with external light sources able to emit modulated light. The properties of an RVS (e.g., frequency, color) depend on the rendering device and influence the SSVEP characteristics. This affects the BCI information throughput and the levels of user safety and comfort. Literature on SSVEP-based BCIs does not generally provide reasons for the selection of the used rendering devices or RVS properties. In this paper, we review the literature on SSVEP-based BCIs and comprehensively report on the different RVS choices in terms of rendering devices, properties, and their potential influence on BCI performance, user safety and comfort.

## 1. Introduction

A brain-computer interface (BCI) is a communication system in which the user's intention is conveyed to the external world without involving the normal output pathways of peripheral nerves and muscles [1]. BCIs are especially relevant for users with reduced motor abilities. Yet, applications for a wider range of users are emerging for entertainment, safety, and security.

In noninvasive BCIs, electroencephalography (EEG) is commonly employed because of its high time resolution, ease of acquisition, and cost effectiveness as compared to other brain activity monitoring modalities. Noninvasive electrophysiological sources for BCI control include event-related synchronization/desynchronization (ERS/ERD), visual evoked potentials (VEP), steady-state visual evoked potentials (SSVEP), slow cortical potentials (SCP), P300 evoked potentials and *μ* and *β* rhythms [2]. SSVEP-based BCIs have received increased attention because they can provide relatively higher bit rates of up to 70 bits/min while requiring little training [3].

**Figure 1:**
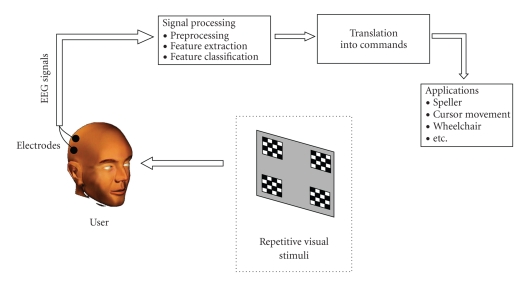
Functional model of an SSVEP-based BCI.

**Figure 2:**
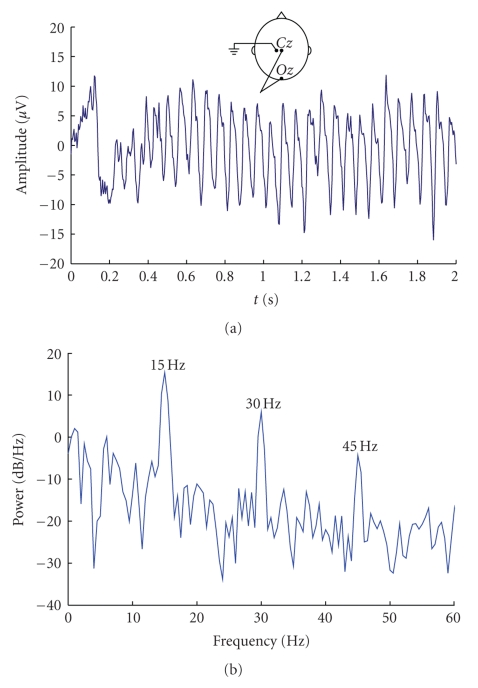
Typical waveform of an EEG signal (Oz-Cz) acquired during visual light stimulation with a frequency of 15 Hz and its frequency spectrum. (a) SSVEP waveform resulting from the time-locked average of 10 realizations. A transient VEP can be observed at the moment where the stimulation began and a clear oscillation (the steady state VEP) can be seen afterwards; (b) Frequency content of the signal in (a). The SSVEP manifests itself in oscillations at 15 Hz and higher harmonics.

An SSVEP-based BCI (see the functional model in Figure 1) enables the user to select among several commands that depend on the application, for example, moving a cursor on a computer screen. Each command is associated with a repetitive visual stimulus (RVS) that has distinctive properties (e.g., frequency or phase). The stimuli are simultaneously presented to the user who selects a command by focusing his/her attention on the corresponding stimulus. When the user focuses his/her attention on an RVS, an SSVEP is elicited which manifests as oscillatory components in the user's EEG, especially in the signals from the primary visual cortex, matching the frequency or harmonics of that RVS (see Figure 2). SSVEPs can be elicited by repetitive visual stimuli at frequencies in the 1 to 100 Hz range [4].

SSVEPs can be automatically detected through a series of signal processing steps including preprocessing (e.g., band-pass filtering), artifact detection/correction, feature extraction (e.g., spectral content at the stimulation frequencies), and feature classification. BCI performance is usually assessed in terms of classification accuracy, classification speed, and the number of available choices. These can be aggregated into a single indicator, namely the bit rate [1, 5]. In SSVEP-based BCIs, the classification accuracy is primarily influenced by the strength of the SSVEP response, the signal-to-noise ratio (SNR), and the differences in the properties of the stimuli. The classification speed depends on the time it takes for the SSVEP to be of sufficient strength. Increasing the number of targets offers a higher number of possible commands but can decrease classification accuracy and speed.

In addition to the bit rate, it is also important to consider the safety and comfort of SSVEP-based BCIs. Repetitive visual stimuli modulated at certain frequencies can provoke epileptic seizures [6] and flashes that are excessively bright may impair the user's vision. Furthermore, certain stimulation frequencies can induce fatigue.

The nature of the RVS in an SSVEP-based BCI influences the performance in terms of bit rate and can also have repercussions on user comfort and safety. In spite of being such an essential element of SSVEP-based BCIs, RVS selection is only superficially addressed in most SSVEP publications. Existing review papers focus on general VEP-based BCIs [7] and signal processing algorithms applied to BCIs [2]. This paper reviews the stimuli that have been used for SSVEP-based BCIs with the goals of: (1) categorizing the stimulation strategies reported in literature, and (2) providing a reference document to motivate the stimulus selection for BCI applications.

This paper is organized as follows. Section 2 describes the types of repetitive visual stimuli. Section 3 presents the methods used to conduct the literature survey as well as the inclusion criteria. A detailed categorization of currently used RVS is presented in Section 4. The results are discussed in Section 5 and the conclusions are presented in Section 6.

## 2. Repetitive Visual Stimuli

In SSVEP research, three main categories of repetitive visual stimuli exist.

*Light* stimuli are rendered using light sources such as LEDs, fluorescent lights, and Xe-lights, which are modulated at a specified frequency. These devices are generally driven by dedicated electronic circuitry which enables them to accurately render any illumination sequence or waveform. The intensity (time integrated luminance) of the light stimulus is measured in photopic candela seconds per square meter (*c* *d*·*s*·*m* ^−2^ or *n* *i* *t* *s*·*s*) because the light luminance changes over time, whereas the background luminance is measured in candela per square meter (*c* *d*·*m* ^−2^ or *n* *i* *t* *s*) [8]. An important parameter to quantify the stimulus strength is the modulation depth which is defined as (*l*_max _ − *l*_min _)/(*l*_max _+ *l*_min _), where *l*_min _, *l*_max _ are the minimum and maximum luminance, respectively.

**Figure 3:**
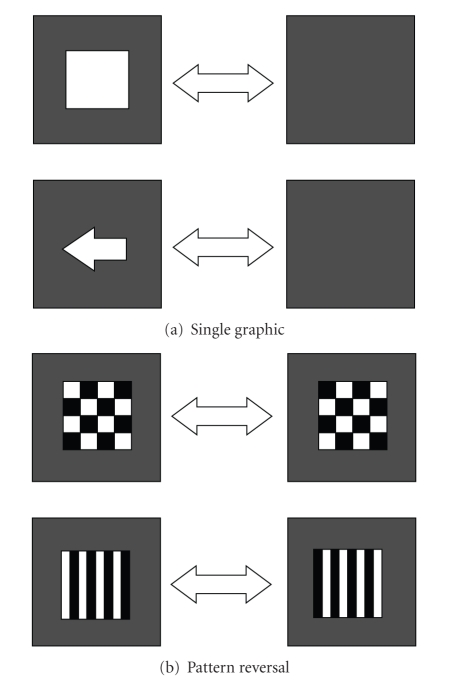
(a) In single graphic stimuli the graphical object alternately appears and disappears in the background. (b) In pattern reversal stimuli at least two patterns are alternated at a specified frequency.

*Single graphics* stimuli (e.g., rectangle, square, or arrow) are rendered on a computer screen and appear from and disappear into the background at a specified rate (see Figure 3(a)).The stimulation rate is reported as the number of full cycles per second, normally simply referred to as the frequency of the stimulus.

*Pattern reversal* stimuli are rendered on a computer screen by oscillatory alternation of graphical patterns, for example, checkerboards. They consist of at least two patterns that are alternated at a specified number of alternations per second [8]. Frequently used patterns include checkerboards and lineboxes (see Figure 3(b)). Patterns are usually colored in black and white. A checkerboard stimulus is characterized by the subtended visual angle of each tile (spatial frequency), the number of reversals per second, the mean luminance, the field size, and the pattern contrast.

It is worth noting that single graphic stimuli could be viewed as a special case of pattern reversal stimuli where the graphic is the first pattern and the second pattern is the background. An important difference is that single graphic stimuli elicit an SSVEP response at the frequency of one full cycle (i.e. two alternations), whereas real pattern reversal stimuli elicit an SSVEP response at the frequency of one alternation.

All repetitive visual stimuli have various properties such as frequency, color, and contrast. Both the type and properties of stimuli affect the elicited SSVEP response.

## 3. Literature Search and Inclusion Criteria

**Figure 4:**
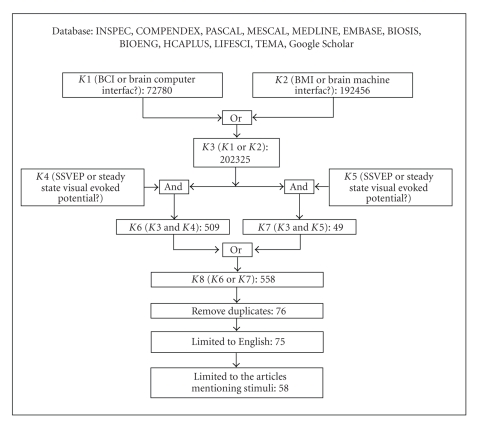
Literature search strategy and the number of papers retrieved at each step. “K” indicates “keyword” and “?” refers to arbitrary characters (e.g., e or es).

To conduct the literature survey on the stimulation strategies in SSVEP-based BCIs, the following databases were consulted: INSPEC, COMPENDEX, PASCAL, MESCAL, MEDLINE, EMBASE, BIOSIS, BIOENG, HCAPLUS, LIFESCI, TEMA, and Google Scholar. Papers were selected for review if the following classes of terms are present in their title, abstract or keyword list: (1)*BCI*, *Brain-Computer Interfac?*, *BMI* and *Brain Machine Interfac?*; (2)*SSVEP*, *Steady State Visual Evoked Potential?*, *SSVER* and *Steady State Visual Evoked Respons?*; where the question mark “*?*” represents arbitrary letters (e.g., “e”, “es” or “ing”). Figure 4 illustrates the search strategy as well as the number of papers retrieved at each step.

To be included in the review, papers had to distinctly mention the used stimulus. Papers that used SSVEP to research the visual pathway and attention as opposed to the goal of building BCI systems were excluded. Only papers written in English prior to June 2009 were considered.

## 4. State of the Art

Fifty-seven papers met the inclusion criteria. They are categorized into three classes according to the type of RVS they use: light, single graphic, and pattern reversal stimuli. Tables 1, 2, and 3 detail the specific properties of the RVS associated with the three classes.

In the remainder of this article we mainly consider the rendering devices, stimulation frequencies, and colors. The rendering device can significantly affect the strength of the SSVEP signal [9]. The stimulation frequency is an important property of the RVS. All the BCI systems reviewed in this paper use stimulation frequencies in the 4 to 50 Hz range. In [10] these frequencies were classified into three frequency bands: low (1–12 Hz), medium (12–30 Hz), and high (30–60 Hz). In each table, these three bands are used to sub-categorize the papers. Stimulus color also influences the SSVEP because the SSVEP responses are different for red, blue, and yellow light [11].

The history of the use of different stimuli in SSVEP-based BCIs is summarized in Tables 1, 2, and 3. The first known SSVEP-based BCI was presented in 1996 [12] and used a fluorescent light to render the stimulation. This system had only one stimulus and was based on the self-regulation of the SSVEP amplitude. Stimuli displayed on computer screens have been used since 1999. Single graphics were used to mimic light stimuli. The graphics included squares or rectangles [13] and arrows [14]. Since then, more than one stimulus were used and each stimulus corresponded to a different command. Although LEDs are popular in current SSVEP-based BCIs, they were not used as rendering devices until 2003 [15]. Pattern reversal is commonly used in transient VEP research and can elicit more prominent VEPs than other stimuli. It was first used in 2004 in an SSVEP-based BCI [16]. For some clinical applications the EEG recording equipment has its own visual stimulation (e.g., Xe-light). This type of stimulation was also tested in [17]. The color of the stimulus was first considered in 2001 [18].

Out of the 58 reviewed papers, 14 use checkerboards, 18 use rectangular stimuli on a computer screen, 1 uses arrows, 1 uses lineboxes, 24 use LEDs, 1 uses a fluorescent light and 1 uses an Xe-light. The sum exceeds the 58 reviewed papers because some employ more than one stimulation method.

The low and medium-frequency bands are both used in 49 of the reviewed articles, while the high-frequency band was only employed in 8. A combination of the low and medium frequency bands is used by 30 of the papers, while 1 uses a combination of the low and high frequency bands, 2 use a combination of the medium and high frequency bands, 1 uses all three frequency bands and 1 does not mention the frequency used.

Slightly more research has been conducted using computer screens than with light stimuli (33 versus 26 articles). More articles feature single graphic stimuli than pattern reversal (19 versus 14 articles). LEDs are almost always used for light stimuli, while plain rectangles and checkerboards are the basic choices for single graphic and pattern reversal stimuli. Other choices are rarely used [12, 14, 17, 62].

For stimulation on computer monitors, mostly black, and white colors are used. For light stimuli the colors red, white and green are frequently used. It is worth noticing that the two best-performing BCIs in this category used green lights [3, 15]. Further research on the influence of color on the SSVEP is necessary.

Direct comparison of the performance of different stimuli based on the performance of the BCIs that employed them is difficult due to the large number of variables that may influence a BCI's performance in addition to the stimulation properties. Furthermore, a large inter-subject variability of SSVEP response exists. However, such a comparison can still provide an indication on how suitable different stimuli are for BCI. We therefore list the best and median performance of SSVEP-based BCIs using LEDs, checkerboards, and squares here to give an indication: a system using LEDs achieved a bit rate of 68 bits/min with 48 choices [15], a pattern reversal system reached a bit rate of 45.5 bits/min with 8 choices [57], and a system using rectangle stimuli obtained a bit rate of 58 bits/min with 6 choices [45]. The median bit rate for systems using LED stimulation is 42 bits/min, while for single graphics it is 35.075 bits/min and pattern reversal systems achieve 26 bits/min. Unfortunately most articles either did an offline analysis or failed to mention the performance of the presented BCI systems in terms of bit rate.

In addition to the bitrate, user safety and comfort are important for the commercial applicability of SSVEP-based BCIs. However, these aspects are very rarely mentioned in the literature.

## 5. Discussion

In this section we first discuss the effect of the repetitive visual stimuli that are regularly used in the reviewed literature on the SSVEP. We then present innovative stimulation designs that were designed to address some of the most relevant issues in BCI such as preventing loss of attention during operation, increasing the number of stimuli, SNR enhancement, and independent operation.

### 5.1. RVS Effect on SSVEP

Stimulation type, frequency, and color have all an effect on the SSVEP response they elicit.

#### 5.1.1. Stimulation Type

The reviewed papers were categorized into three tables according to whether they used light, single graphic, or pattern reversal stimuli. The SSVEP response to these three types of stimuli is different. Pattern reversal stimuli can produce a more pronounced SSVEP than single graphic stimuli modulated at the same frequency [56]. In [9] light and single graphic stimuli were generated at 4.6, 10.8, and 16.1 Hz. It was found that the SSVEP response elicited by an LED was larger than that by a rectangle stimulus on a computer screen. Also it was stated that the SSVEP response for light stimuli was larger than that for pattern reversal in [10]. This might explain why we found that the bit rates of BCIs using LED stimuli appear to be higher compared to those of BCIs using computer screens. For each of these results, most variables were fixed (e.g., luminance, contrast, and color). At present, no general conclusions can be drawn because many conditions have not been tested and variables can interact with each other. For instance, the power of the SSVEP response is affected by both frequency and color of the stimuli [11].

From the viewpoint of implementation, it is in general easier to build a BCI that employs a computer screen as it mainly relies on software development and no hardware modification is necessary. Furthermore, BCI designers are completely free in their choice of development platform for the implementation of this software. Use of computer monitors offers flexibility for combining BCI stimulation with the controlled application and makes it possible for the stimulation interface to easily be fine-tuned during BCI development or even for it to change during a BCI session.

BCIs using light stimuli on the other hand usually require the development of dedicated hardware in addition to software. Also, the used hardware often restricts the number of development platforms that can be used for software development. In return for this investment comes an extreme flexibility in the signals and frequencies that can be generated, because LEDs are usually controlled by waveform generators that are capable of generating many different frequencies. LEDs are said to be preferable in practical applications that require more than 20 choices, because monitors have difficulties to accurately display various stimuli at different frequencies [9].

Using a monitor severely limits the range of frequencies that can be used for stimulation. The refresh rate *R* of the monitor, that is, the number of times that the monitor redraws the screen per second, is usually lower than 100 Hz (for LCD monitors it is usually 60 Hz). Only frequencies that are lower than *R*/2 Hz can be used [67] and only the subharmonics of the screen refresh rate can be obtained [50]. Errors appear when rendering frequencies whose periods are not multiples of 2/*R*. Such frequencies are either very low to elicit an SSVEP or are each others harmonics. This is often undesirable for SSVEP-based BCIs. Because of this, these BCIs often use frequencies that can be displayed less accurately. The rendering of the frequency can be further hindered by the task scheduling that most operating systems perform, which can cause unpredictable delays. Finally, if a large number of target stimuli have to be used, the computational load of generating or displaying them may cause inaccuracies in the displayed stimulations.

Computer screens with higher refresh rates exist (e.g., a screen refreshing at 120 Hz used in [59]), but are increasingly difficult to obtain commercially. Such screens can increase the available number of frequencies, but do not solve the above problem completely.

#### 5.1.2. Stimulus Frequency

As mentioned in Section 4, the stimulus frequencies used in SSVEP research can be classified into three frequency bands, that is, low (1–12 Hz), medium (12–30 Hz) and high (30–60 Hz). The largest SSVEP amplitudes were observed near 10 Hz followed by 16–18 Hz and the high frequency subsystem showed the smallest response [10]. As shown in Tables 1, 2, and 3, many SSVEP-based BCIs used the low and medium frequency bands, although the frequencies varied significantly. These two frequency bands, however, have some disadvantages. First, subjective evaluations showed that frequencies between 5 and 25 Hz are more annoying than higher ones; visual fatigue would easily occur. Second, flash and pattern reversal stimuli can provoke epileptic seizures especially in the 15–25 Hz range [6]. Third, the low frequency band covers the alpha band (8–13 Hz) which can cause a considerable amount of false positives. All of these disadvantages can be avoided by using the high frequency band.

The disadvantage of a weak SSVEP response is mitigated by the fact that there is less spontaneous brain activity in the high frequency band compared to lower ones [46]. Additionally, spatial filters that combine several lead signals into one channel [34] can be used to increase the SSVEP energy enough so it can effectively be used in a BCI. Furthermore, the SNR of the SSVEP response (calculated as the ratio of EEG power at the stimulation frequency to the mean power of the adjacent frequency bands) is similar in all frequency bands [46]. An offline analysis showed that utilizing the high frequency band can be very promising [38]. Therefore, the high frequency band can be expected to be applied in SSVEP-based BCIs in the future and should definitely be researched further.

#### 5.1.3. Stimulus Color

It was reported in [11] that red, yellow, and blue light stimuli have different effects on the SSVEP in combination with the used frequency. Red light elicited the strongest response when modulated at 11 Hz, but SSVEP strength went downhill fast for surrounding frequencies. Blue light stimuli elicited a slightly weaker strongest response around 13 Hz, but were less sensitive to the used frequency. The SSVEP strength elicited by yellow light was lower and less dependent on the used frequency. Another study that focused on stimulus color showed that the second and fourth harmonic of the SSVEP are affected differently by chromatic and achromatic checkerboard stimuli [68].

At present, green, red, gray, black, and white stimuli have been used for SSVEP-based BCIs. It is difficult to decide which color is the best, because at present there is no comparison that shows how color influences the performance of SSVEP-based BCIs. A good solution for practical applications could be to use stimuli whose colors can be dynamically adjusted in order to take circumstances or the user's characteristics into account.

### 5.2. Stimuli Improvements

Recent studies present some new stimulus designs based on more standard stimulation methods. Four important goals to be achieved with these enhancements are: (1) to maximize selective attention and to minimize the eye movements with respect to the controlled element; (2) to increase the number of available frequencies; (3) to enhance the SSVEP SNR; and (4) to change an SSVEP-based BCI from dependent to independent.

#### 5.2.1. Maintaining Attention on the Stimuli

The position of the stimuli in current SSVEP-based BCIs is often fixed. However, the user needs feedback during BCI operation. While the user is moving an element (e.g., a cursor or a virtual car), his/her eyes can occasionally move away from the stimuli. Furthermore, the user can be distracted, which can deteriorate the signal because the SSVEP strength is strongly influenced by attention [69]. A possible solution for mitigating this problem is to make the stimuli move along with the controlled elements. In [57, 60], the stimulation unit was designed as a smart multiple choice table in the form of an array of small checkerboard images moving along with the controlled elements and was applied to a real-time BCI with a bit rate higher than 26 bits/min.

#### 5.2.2. Increase the Number of Available Frequencies

Most current SSVEP-based BCIs use one frequency per target. Hence a large number of targets require a large number of frequencies. However, the frequency range with relatively high SSVEP responses is limited. Increasing the number of targets then decreases the frequency resolution which in turn makes classification more difficult. This is especially problematic on computer screens, since we have difficulty generating all but a select few frequencies accurately.

One solution is to differ the relative phases of the stimuli so that phase information can also be used to distinguish among targets. In [7, 54], all stimuli flickered at the same frequency and differed only in relative phase.

A second solution attempts to mitigate the problem by using dual-frequency stimulation: modulating a single stimulus with two frequencies. By adding together two frequencies *F*_1_ and *F*_2_ = *F*_1_/2 a third stimulus *F*_1_+*F*_2_ was obtained which would evoke peaks in the SSVEP signal at *F*_1_, *F*_2_, *F*_1_ + *F*_2_ and their harmonics [64]. Thus three options could be obtained using only two frequencies. In [64], the stimulus was a checkerboard rendered on a computer screen. This solution can also be applicable with light sources such as LEDs.

Unfortunately, these solutions have only been evaluated with two or three targets and were so far not tested thoroughly in online systems in which many targets exist.

#### 5.2.3. Enhance the SSVEP SNR

High SSVEP SNR can simplify the feature extraction and improve the classification accuracy. In [27], a novel method based on half-field alternate stimulation was proposed to enhance the SSVEP SNR. The optic nerves from the retina's left and right halves cross at the so-called optic chiasm and finally reach the left and right part of the primary visual cortex. Based on this, a target stimulus consisting of two light sources that flashed with the same frequency but opposite in phase was proposed. Because the light sources flashed at different times and were located in different parts of the visual field the workload of the left and the right part of the primary visual cortex was alternated. Subtracting the signals obtained at the left and right occipital lobes from one another suppressed the noise from muscle-originated signals and spontaneous brain waves, and thereby enhanced the SSVEP SNR.

#### 5.2.4. From Dependent to Independent

According to the definition of [1], BCIs can be either dependent or independent. A dependent BCI requires some activity from the brain's normal output pathways (e.g., muscles), while an independent BCI does not depend in any way on these output pathways. SSVEP-based BCIs are generally considered as dependent, because the user has to change his gaze direction to focus on the desired target. This might not work if the user is so severely disabled that he is unable to reliably control gaze. Consequently, it is very useful to make an independent SSVEP-based BCI. In order to make this improvement, one attractive option is to develop a stimulus which is able to evoke different SSVEP responses without the user's gaze.

The BCI in [49] utilized electrophysiological correlates of visual spatial attention mechanisms to make binary selection of left and right visual targets. Besides spatial attention, another solution is selectively paying attention to a certain stimulation of an overlapping stimulus. Two superimposed images consisting of vertical and horizontal parallel bars flickering at different frequencies were presented [62, 70]. A similar stimulus design was used in [18], where a red/black and green/black square alternating at different frequencies were superimposed on each other and yellow was used when both stimuli were in the “on” state. In another study spatially intermingled red and blue motion dots flickered at different frequencies while continuously shifting their positions at random [71]. All of these methods are based on the fact that selective attention to one stimulus while ignoring the other will enhance the amplitude of the SSVEP of the attended frequency [72].

## 6. Conclusion

SSVEP-based BCIs allow users to communicate with the external world by selectively paying attention to one out of a set of repetitive visual stimuli. In this review, we have highlighted important facts of these stimuli in BCIs: (1) checkerboard, rectangle, and LED-based stimulation are the most frequently used stimulation types, (2) stimulation frequencies in the low and medium frequency bands have been more often applied than those in the high frequency band even though the latter offer higher levels of comfort and safety.

From the reported bit rates it appears that SSVEP-based BCIs that use LEDs for stimulation have higher bit rates (median 42 bits/minute) than those using computer screens that render the stimuli through single graphic alternation (median 35.075 bits/minute) or pattern reversal (median 26 bits/minute). For a small number of RVS both computer screens and LEDs are plausible as rendering devices. For a large number of RVS (more than 20 according to [9]) or stimulation frequencies in the high frequency band, LEDs are preferable.

The choice of properties of the used stimuli can affect the performance, safety, and comfort of an SSVEP-based BCI. Improvements to stimuli can enhance the SSVEP SNR, simplify signal processing, enable the use of more targets, prevent loss of attention, and allow for BCI independent BCI operation.

## Figures and Tables

**Table 1:** Characteristics of light stimuli.

Frequency band	Study	Stimulus			Bit rate (bits/min)
		Device	Frequency (Hz)	Color	
L	Maggi et al. 2006 [19]	LED	6, 7, 8, 10 Hz	Green	—
	Piccini et al. 2005 [20]	LED	6–10 Hz	—	—

M	Lüth et al. 2007 [21]	LED	13, 14, 15, 16, 17 Hz	Red	—
	Valbuena et al. 2007 [22]	LED	13, 14, 15, 16 Hz	—	—
	Leow et al. 2007 [23]	LED	14–29 Hz	Red	—
	Materka and Byczuk 2006 [24]	LED	25, 26.5625, 28.125, 29.6875 Hz	—	—
	Calhoun et al. 1996 [12]	Fluorescent light	13.25 Hz	—	—

H	Garcia Molina 2008 [25]	LED	40–50 Hz	White	—
	Huang et al. 2008 [17]	Xe-light	30–50 Hz	—	—
	Materka et al. 2007 [26]	LED	32–40 Hz	—	—
	Materka and Byczuk, 2006 [27]	LED	34–40 Hz	—	—

L+M	Parini et al. 2009 [3]	LED	6, 7,…, 17 Hz	Green	51.47
	Bin et al. 2008 [28]	LED	10, 11, 12, 13 Hz	—	—
	Wu and Yao 2008 [29]	LED	8.3, 10 Hz	White	—
	Wu et al. 2008 [9]	LED	4.6, 10.8, 16.1 Hz	White	—
	Müller-Putz et al. 2008 [30]	LED	6, 7, 8, 13 Hz	Red	—
	Müller-Putz and Pfurtschelle 2008 [31]	LED	6, 7, 8, 13 Hz	Red	—
	Scherer et al. 2007 [32]	LED	6.25, 7.25, 8.00, 13.00 Hz; 11.75, 13.00, 15.25, 17.25 Hz	Red	—
	Jia et al. 2007 [33]	LED	6, 6.5, 7,…, 19 Hz	—	46.1
	Friman et al. 2007 [34]	LED	5, 7, 9, 11, 13, 15 Hz	—	—
	Friman et al. 2007 [35]	LED	13, 14, 15, 16, 17 Hz	Red	27–30
	Müller-Putz et al. 2005 [36]	LED	6, 7, 8, 13 Hz	Red	31.5
	Wang et al. 2004 [37]	LED	9–17 Hz	—	42
	Gao et al. 2003 [15]	LED	6, 6.195, 6.390,…, 15	Green	68

M+H	Wang et al. 2005 [38]	LED	21, 23,…, 43 Hz	White	—

L+M+H	Ruen et al. 2007 [39]	LED	7–35 Hz	Red	—

**Table 2:** Characteristics of single graphic stimuli.

Frequency band	Study	Stimulus				Bit rate (bits/min)
		Device	Shape	Frequency (Hz)	Color	
L	Wang et al. 2008 [7]	—	Square	10 Hz	—	—
	Ren et al. 2008 [40]	—	Square	10 Hz	White/black	—
	Touyama and Hirose, 2007 [41]	—	Cube	4.80, 6.86 Hz	—	—
	Touyama and Hirose, 2007 [42]	—	Cube	4.80, 6.86 Hz	—	—
	Beverina et al. 2003 [14]	—	Arrow	6, 10 Hz	Green	—
	Cheng and Gao, 1999 [13]	—	Block	6–9 Hz	—	—

M	Cecotti and Graeser, 2008 [43]	LCD	Box	15.5, 16,…, 17.5 Hz	—	—
	Kelly et al. 2005 [44]	CRT	Rectangle	14, 17 Hz	White/black	7.5

L+M	Bin et al. 2009 [45]	LCD	Square	6.5, 7.5, 8.6, 10, 12, 15 Hz	White/black	58
	Wu et al. 2008 [9]	LCD and CRT	Square	4.6, 10.8, 16.1 Hz	White/black	—
	Wang et al. 2006 [46]	CRT	Button	9–17 Hz	—	43
	Nielsen et al. 2006 [47]	CRT	Square	5.0, 7.08, 7.73, 8.5, 9.44, 10.63, 12.14, 14.16, 17.0 Hz	—	21
	Kelly et al. 2005 [48]	CRT	Rectangle	9.45, 10.63 Hz; 14.17, 17.01 Hz	White/black	—
	Kelly et al. 2005 [49]	CRT	Rectangle	10.03, 12.04 Hz	White/black	—
	Wahnoun et al. 2002 [50]	—	Block	5.000, 7.080, 7.727, 8.927, 11.087, 12.140, 12.750, 17.000, 21.250 Hz	White and a small light gray in the middle	—
	Cheng et al. 2002 [51]	CRT	Button	6–14 Hz	—	27.15
	Cheng et al. 2001 [18]	—	Block	6.45, 7.23, 8.01, 13.87 Hz	Red, green, and yellow	—

L+H	Sami and Nielsen, 2004 [52]	CRT	Rectangle	8.8, 35 Hz	—	—

M+H	Lin et al. 2007 [53]	CRT	Squares	27,29,…, 43 Hz	—	—

**Table 3:** Characteristics of pattern reversal stimuli.

Frequency band	Study	Stimulus				Bit rate (bits/min)
		Device	Shape	Frequency (Hz)	Color	
L	Kluge and Hartmann,2007 [54]	TFT	Checkerboard	10, 12 Hz	—	—
	Trejo et al. 2006 [55]	LCD	Checkerboard	5, 5.625, 6.4, 6.9 Hz	White/black	—
	Lalor et al. 2005 [56]	—	Checkerboard	8.5, 10 Hz	White/black	10.3

M	Kelly et al. 2004 [16]	—	Checkerboard	17, 20 Hz	White/black	—

L+M	Vasquez et al. 2008 [57]	CRT	Checkerboard	8.8, 9.4, 11.55, 12.5, 13.65, 15, 16.7, 18.8 Hz	White/black	45.5
	Oehler et al. 2008 [58]	—	Checkerboard	10–15 Hz	White/black	12.5
	Martinez et al. (2008 [59], 2007 [60])	CRT	Checkerboard	5, 6, 7, 8 Hz; 12, 13.3, 15, 17 Hz	White/black	26–30
	Krusienski and Allison, 2008 [61]	—	Checkerboard	6, 15 Hz	White/black	—
	Allison et al. 2008 [62]	CRT	Lineboxes and checker-board	6, 15 Hz	White/black; gray/white; red/gray; green/gray	—
	Bakardjian et al. 2007 [63]	—	Checkerboard	8, 12, 14, 28 Hz	White/black	—
	Mukesh et al. 2006 [64]	—	Checkerboard	6, 7, 12, 13, 14 Hz	White/black	—
	Jaganathan et al. 2005 [65]	—	Checkerboard	6–15 Hz	White/black	—

—	Lalor et al. 2004 [66]	—	Checkerboard	—	White/black	—

## References

[1] Wolpaw JR, Birbaumer N, McFarland DJ, Pfurtscheller G, Vaughan TM (2002). Brain-computer interfaces for communication and control. *Clinical Neurophysiology*.

[2] Bashashati A, Fatourechi M, Ward RK, Birch GE (2007). A survey of signal processing algorithms in brain-computer interfaces based on electrical brain signals. *Journal of Neural Engineering*.

[3] Parini S, Maggi L, Turconi AC, Andreoni G (2009). A robust and self-paced BCI system based on a four class SSVEP paradigm: algorithms and protocols for a high-transfer-rate direct brain communication. *Computational Intelligence and Neuroscience*.

[4] Herrmann CS (2001). Human EEG responses to 1–100 Hz flicker: resonance phenomena in visual cortex and their potential correlation to cognitive phenomena. *Experimental Brain Research*.

[5] Pun T, Alecu TI, Chanel G, Kronegg J, Voloshynovskiy S (2006). Brain-computer interaction research at the Computer Vision and Multimedia Laboratory, University of Geneva. *IEEE Transactions on Neural Systems and Rehabilitation Engineering*.

[6] Fisher RS, Harding G, Erba G, Barkley GL, Wilkins A (2005). Photic- and pattern-induced seizures: a review for the epilepsy foundation of america working group. *Epilepsia*.

[7] Wang Y, Gao X, Hong B, Jia C, Gao S (2008). Brain-computer interfaces based on visual evoked potentials: feasibility of practical system designs. *IEEE Engineering in Medicine and Biology Magazine*.

[8] Odom JV, Bach M, Barber C (2004). Visual evoked potentials standard (2004). *Documenta Ophthalmologica*.

[9] Wu Z, Lai Y, Xia Y, Wu D, Yao D (2008). Stimulator selection in SSVEP-based BCI. *Medical Engineering and Physics*.

[10] Regan D (1989). *Human Brain Electrophysiology: Evoked Potentials and Evoked Magnetic Fields in Science and Medicine*.

[11] Regan D (1966). An effect of stimulus colour on average steady-state potentials evoked in man. *Nature*.

[12] Calhoun GL, McMillan GR EEG-based control for human-computer interaction.

[13] Cheng M, Gao S An EEG-based cursor control system.

[14] Beverina F, Palmas G, Silvoni S, Piccione F, Giove S (2003). User adaptive BCIs: SSVEP and P300 based interfaces. *PsychNology Journal*.

[15] Gao X, Xu D, Cheng M, Gao S (2003). A BCI-based environmental controller for the motion-disabled. *IEEE Transactions on Neural Systems and Rehabilitation Engineering*.

[16] Kelly SP, Lalor E, Finucane C, Reilly RB A comparison of covert and overt attention as a control option in a steady-state visual eyoked potential-based brain computer interface.

[17] Huang M, Wu P, Liu Y, Bi L, Chen H Application and contrast in brain-computer interface Between hilbert-huang transform and wavelet transform.

[18] Cheng M, Gao X, Gao S, Xu D Multiple color stimulus induced steady state visual evoked potentials.

[19] Maggi L, Parini S, Piccini L, Panfili G, Andreoni G A four command BCI system based on the SSVEP protocol.

[20] Piccini L, Parini S, Maggi L, Andreoni G A wearable home BCI system: preliminary results with SSVEP protocol.

[21] Lüth T, Ojdanić D, Friman O, Prenzel O, Gräser A Low level control in a semi-autonomous rehabilitation robotic system via a brain-computer interface.

[22] Valbuena D, Cyriacks M, Friman O, Volosyak I, Gräser A Brain-computer interface for high-level control of rehabilitation robotic systems.

[23] Leow RS, Ibrahim F, Moghavvemi M Development of a steady state visual evoked potential (SSVEP)-based brain computer interface (BCI) system.

[24] Materka A, Byczuk M Using comb filter to enhance SSVEP for BCI applications.

[25] Garcia Molina G (2008). High frequency SSVEPs for BCI applications. *Brain-Computer Interfaces for HCI and Games*.

[26] Materka A, Byczuk M, Poryzala P A virtual keypad based on alternate half-field stimulated visual evoked potentials.

[27] Materka A, Byczuk M (2006). Alternate half-field stimulation technique for SSVEP-based brain-computer interfaces. *Electronics Letters*.

[28] Bin G, Lin Z, Gao X, Hong B, Gao S The SSVEP topographic scalp maps by canonical correlation analysis.

[29] Wu Z, Yao D (2008). Frequency detection with stability coefficient for steady-state visual evoked potential (SSVEP)-based BCIs. *Journal of Neural Engineering*.

[30] Müller-Putz GR, Eder E, Wriessnegger SC, Pfurtscheller G (2008). Comparison of DFT and lock-in amplifier features and search for optimal electrode positions in SSVEP-based BCI. *Journal of Neuroscience Methods*.

[31] Müller-Putz GR, Pfurtscheller G (2008). Control of an electrical prosthesis with an SSVEP-based BCI. *IEEE Transactions on Biomedical Engineering*.

[32] Scherer R, Müller-Putz GR, Pfurtscheller G (2007). Self-initiation of EEG-based brain-computer communication using the heart rate response. *Journal of Neural Engineering*.

[33] Jia C, Xu H, Hong B, Gao X, Zhang Z, Gao S A human computer interface using SSVEP-based BCI technology.

[34] Friman O, Volosyak I, Gräser A (2007). Multiple channel detection of steady-state visual evoked potentials for brain-computer interfaces. *IEEE Transactions on Biomedical Engineering*.

[35] Friman O, Lüth T, Volosyak I, Gräser A Spelling with steady-state visual evoked potentials.

[36] Müller-Putz GR, Scherer R, Brauneis C, Pfurtscheller G (2005). Steady-state visual evoked potential (SSVEP)-based communication: impact of harmonic frequency components. *Journal of Neural Engineering*.

[37] Wang Y, Zhang Z, Gao X, Gao S Lead selection for SSVEP-based brain-computer interface.

[38] Wang Y, Wang R, Gao X, Gao S Brain-computer interface based on the high-frequency steady-state visual evoked potential.

[39] Ruen SL, Ibrahim F, Moghavvemi M Assessment of steady-state visual evoked potential for brain computer communication.

[40] Ren R, Bin G, Gao X Idle state detection in SSVEP-based brain-computer interfaces.

[41] Touyama H, Hirose M Brain computer interface via stereoscopic images in CAVE.

[42] Touyama H, Hirose M Steady-state VEPs in CAVE for walking around the virtual world.

[43] Cecotti H, Graeser A Convolutional neural network with embedded fourier transform for EEG classification.

[44] Kelly SP, Lalor E, Reilly RB, Foxe JJ Independent brain computer interface control using visual spatial attention-dependent modulations of parieto-occipital alpha.

[45] Bin G, Gao X, Yan Z, Hong B, Gao S (2009). An online multi-channel SSVEP-based brain-computer interface using a canonical correlation analysis method. *Journal of Neural Engineering*.

[46] Wang Y, Wang R, Gao X, Hong B, Gao S (2006). A practical VEP-based brain-computer interface. *IEEE Transactions on Neural Systems and Rehabilitation Engineering*.

[47] Nielsen KD, Cabrera AF, do Nascimento OF (2006). EEG based BCI—towards a better control. Brain-computer interface research at Aalborg University. *IEEE Transactions on Neural Systems and Rehabilitation Engineering*.

[48] Kelly SP, Lalor EC, Reilly RB, Foxe JJ (2005). Visual spatial attention tracking using high-density SSVEP data for independent brain-computer communication. *IEEE Transactions on Neural Systems and Rehabilitation Engineering*.

[49] Kelly SP, Lalor EC, Finucane C, McDarby G, Reilly RB (2005). Visual spatial attention control in an independent brain-computer interface. *IEEE Transactions on Biomedical Engineering*.

[50] Wahnoun R, Saigal R, Gu Y A realtime brain-computer interface based on steady-state visual evoked potentials.

[51] Cheng M, Gao X, Gao S, Xu D (2002). Design and implementation of a brain-computer interface with high transfer rates. *IEEE Transactions on Biomedical Engineering*.

[52] Sami S, Nielsen KD Communication speed enhancement for visual based brain computer interfaces.

[53] Lin Z, Zhang C, Wu W, Gao X (2007). Frequency recognition based on canonical correlation analysis for SSVEP-based BCIs. *IEEE Transactions on Biomedical Engineering*.

[54] Kluge T, Hartmann M Phase coherent detection of steady-state evoked potentials: experimental results and application to brain-computer interfaces.

[55] Trejo LJ, Rosipal R, Matthews B (2006). Brain-computer interfaces for 1-D and 2-D cursor control: designs using volitional control of the EEG spectrum or steady-state visual evoked potentials. *IEEE Transactions on Neural Systems and Rehabilitation Engineering*.

[56] Lalor EC, Kelly SP, Finucane C (2005). Steady-state VEP-based brain-computer interface control in an immersive 3D gaming environment. *Eurasip Journal on Applied Signal Processing*.

[57] Vasquez PM, Bakardjian H, Vallverdu M, Cichocki A Fast multi-command SSVEP brain machine interface without training.

[58] Oehler M, Neumann P, Becker M, Curio G, Schilling M Extraction of SSVEP signals of a capacitive EEG helmet for human machine interface.

[59] Martinez P, Bakardjian H, Cichocki A (2008). Multi-command real-time brain machine interface using SSVEP: feasibility study for occipital and forehead sensor locations. *Advances in Cognitive Neurodynamics*.

[60] Martinez P, Bakardjian H, Cichocki A (2007). Fully online multicommand brain-computer interface with visual neurofeedback using SSVEP paradigm. *Computational Intelligence and Neuroscience*.

[61] Krusienski DJ, Allison BZ Harmonic coupling of steady-state visual evoked potentials.

[62] Allison BZ, McFarland DJ, Schalk G, Zheng SD, Jackson MM, Wolpaw JR (2008). Towards an independent brain-computer interface using steady state visual evoked potentials. *Clinical Neurophysiology*.

[63] Bakardjian H, Martinez P, Cichocki A (2007). Dynamic online target control of SSVEP-based brain computer interface with multiple commands. *Neuroscience Research*.

[64] Mukesh TMS, Jaganathan V, Reddy MR (2006). A novel multiple frequency stimulation method for steady state VEP based brain computer interfaces. *Physiological Measurement*.

[65] Jaganathan V, Mukesh TMS, Reddy MR Design and implementation of high performance visual stimulator for brain computer interfaces.

[66] Lalor E, Kelly SP, Finucane C, Burke R, Reilly RB, McDarby G (2004). Brain computer interface based on the steady-state VEP for immersive gaming control. *Biomedizinsche Tecknik*.

[67] Sugiarto I, Allison B, Gräser A Optimization strategy for SSVEP-based BCI in spelling program application.

[68] Arakawa K, Tobimatsu S, Tomoda H, Kira J-I, Kato M (1999). The effect of spatial frequency on chromatic and achromatic steady-state visual evoked potentials. *Clinical Neurophysiology*.

[69] Müller MM, Malinowski P, Gruber T, Hillyard SA (2003). Sustained division of the attentional spotlight. *Nature*.

[70] Chen Y, Seth AK, Gally JA, Edelman GM (2003). The power of human brain magnetoencephalographic signals can be modulated up or down by changes in an attentive visual task. *Proceedings of the National Academy of Sciences of the United States of America*.

[71] Müller MM, Andersen S, Trujillo NJ, Valdés-Sosa P, Malinowski P, Hillyard SA (2006). Feature-selective attention enhances color signals in early visual areas of the human brain. *Proceedings of the National Academy of Sciences of the United States of America*.

[72] di Russo F, Teder-Sälejärvi WA, Hillyard SA (2002). *The Cognitive Electrophysiology of Mind and Brain*.

